# GABA-Producing Bacteria as Potential Psychobiotics in Gut–Brain Axis Regulation

**DOI:** 10.3390/ijms27114969

**Published:** 2026-05-30

**Authors:** Ewelina Zielińska, Katarzyna Kycia, Anna Mikołajczuk-Szczyrba, Natalia Piłka, Edyta Juszczuk-Kubiak

**Affiliations:** 1 Department of Biotechnology, Prof. Wacław Dąbrowski Institute of Agricultural and Food Biotechnology-State Research Institute, Rakowiecka 36 Str., 02-532 Warsaw, Poland; ewelina.zielinska@ibprs.pl (E.Z.); natalia.pilka@ibprs.pl (N.P.); 2 Department of Dairy Biotechnology, Prof. Wacław Dąbrowski Institute of Agricultural and Food Biotechnology-State Research Institute, Rakowiecka 36 Str., 02-532 Warsaw, Poland; 3 Department of Microbiology, Prof. Wacław Dąbrowski Institute of Agricultural and Food Biotechnology-State Research Institute, Rakowiecka 36 Str., 02-532 Warsaw, Poland; anna.mikolajczuk-szczyrba@ibprs.pl

**Keywords:** MGB axis, anxiety, neurotransmitters, GABA, psychobiotics, mental health

## Abstract

γ-Aminobutyric acid (GABA) is the main inhibitory neurotransmitter in the central nervous system (CNS) and plays a vital role in maintaining neural balance, regulating mood, and reducing stress responses. Recent metagenomic studies of the gut microbiome have shown that various bacterial species, especially those in the genera *Lactobacillus*, *Bifidobacterium*, and *Bacteroides*, isolated from the human gut and environmental sources such as fermented foods, contain glutamate decarboxylase (GAD) systems that enable GABA production. Microbially produced GABA can influence the microbiota–gut–brain (MGB) axis by activating neural, endocrine, and immune signalling pathways that are crucial for maintaining gut and brain homeostasis. Emerging evidence suggests that supplementation with GABA-producing bacteria, known as psychobiotics, may improve neurotransmitter balance, modulate cytokine production, strengthen the integrity of the intestinal barrier, and alleviate anxiety- and depression-related behaviours. This review summarises current knowledge of GABA-producing bacterial strains derived from the human gut and food environments and explores their potential as emerging psychobiotics in modulating gut–brain communication and mental health.

## 1. Introduction

The human gastrointestinal tract (GT) hosts a highly diverse and metabolically active community of microorganisms, collectively known as the gut microbiota [1]. It contains trillions of microbial cells, including bacteria, archaea, fungi, and protozoa, as well as many viruses [2]. The composition of the gut microbiota varies along the digestive tract and is influenced by factors such as diet, age, genetics, and environmental conditions [3,4]. In a healthy gut ecosystem, members of the phyla Firmicutes, Bacteroidetes, Actinobacteria, and Verrucomicrobia predominate, and high species diversity underpins stability and resilience against disturbances [5,6]. The gut microbiota plays numerous roles that promote host health. It contributes to nutrient metabolism, supports the immune system, maintains the integrity of the intestinal barrier, and defends against pathogens. Microorganisms inhabiting the GT are crucial for digestion, nutrient absorption, energy harvest, vitamin synthesis, modulation of inflammatory processes, and regulation of host immune responses [3]. The microbiota also influences neurobiological processes via the gut–brain axis, a bidirectional communication system that integrates neural, hormonal, immune, and metabolic signals, which is essential for maintaining systemic homeostasis [7]. Disruption in gut microbiota composition and biodiversity, known as dysbiosis, has been linked to the development of gastrointestinal, metabolic, and neuropsychiatric disorders, including depression and anxiety [8,9]. Dysbiosis leads to compromised intestinal barrier function, persistent inflammatory activation, and disruptions in the production of neuroactive metabolites such as serotonin, dopamine, and GABA, which may contribute to mood disorders and stress-related conditions [8,10,11]. Particular emphasis is placed on GABA, the primary inhibitory neurotransmitter in the central nervous system, responsible for neuronal balance and emotional stability. Beyond its central functions, GABA also regulates gastrointestinal motility and epithelial barrier integrity [12,13,14]. Notably, the gut source of GABA may include lactic acid bacteria (LAB), especially *Lactobacillus* and *Bifidobacterium*, which can produce GABA via the glutamate decarboxylase (GAD) system [15]. This trait supports the concept of psychobiotics—probiotic strains that promote mental health by modulating the gut–brain axis and enhancing GABA production. Growing research suggests that GABA-producing bacterial strains hold therapeutic potential in preventing and treating depression, anxiety, and stress-related illnesses, while serving as a safe adjunct to conventional pharmacological treatments [16,17]. Depression, anxiety, and stress-induced pathologies are among the most significant public health challenges today, impacting individuals and healthcare systems alike. Therefore, the development of new, effective, and safe therapeutic strategies is urgent [17]. In this context, GABA-producing probiotic strains offer a promising avenue of research, as they may help maintain neurochemical balance by influencing the gut–brain axis.

This review synthesises current evidence on GABA-producing bacterial strains from the human gut and food-related environments, highlighting their emerging potential as psychobiotics that modulate gut–brain communication and serve as adjunctive strategies in mental health care.

## 2. Gut Microbiota and Gut–Brain Axis

**Figure 1:**
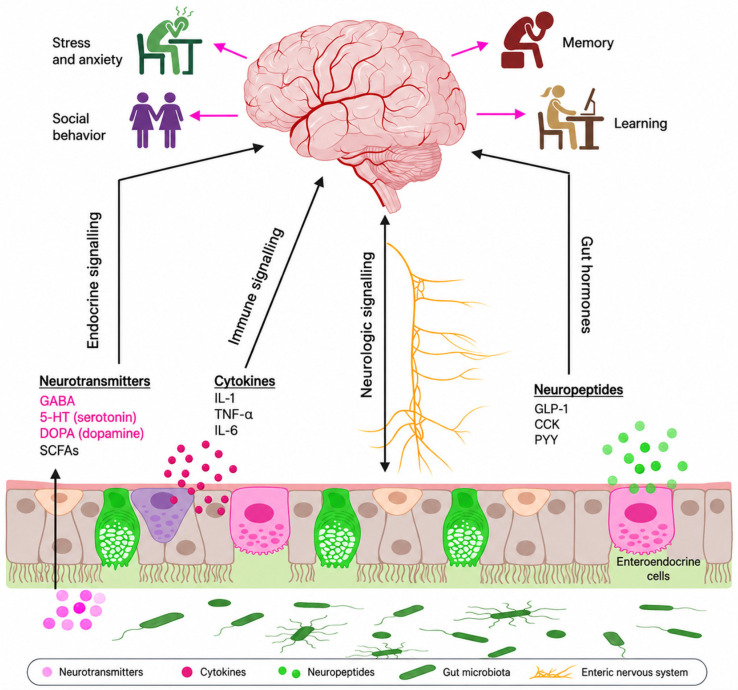
Signalling mechanisms of the microbiota–gut–brain (MGB) axis (created in BioRender (2026) https://BioRender.com/akneihv (accessed on 26 May 2026)).

Recent research indicates that the gut microbiota is closely linked to key aspects of brain function, including cognition, emotional regulation, stress response, and vulnerability to neuropsychiatric disorders [18,19,20]. One of its essential roles is to produce bioactive metabolites, such as short-chain fatty acids (SCFAs) and neuroactive compounds such as indole, serotonin, dopamine, and GABA [21,22]. These metabolites serve as crucial mediators of the two-way communication within the microbiota–gut–brain (MGB) axis, affecting both gastrointestinal health and central nervous system activity [23]. Communication within the MGB axis occurs through three interconnected pathways: neural, immune, and neuroendocrine [24,25] (Figure 1).

The neural pathway involves direct signalling between the gut and the brain via the vagus nerve and the enteric nervous system, allowing microbial metabolites to influence neuronal activity [25,26,27,28,29]. The immune pathway reflects interactions between the gut microbiota and the host immune system, including modulation of cytokine production and regulation of inflammatory responses, which may affect neuroinflammation. The neuroendocrine pathway involves enteroendocrine cells and the hypothalamic–pituitary–adrenal (HPA) axis, linking gut-derived signals with hormonal regulation of stress and brain function [27,30]. A balanced gut microbiota is essential for maintaining the integrity of these communication pathways. In contrast, dysbiosis—characterised by reduced microbial diversity and altered community composition—may disrupt these mechanisms and contribute to the development of gastrointestinal, metabolic, and neuropsychiatric disorders [31,32,33]. Alterations in microbial homeostasis can impair intestinal barrier function, increase gut permeability, and promote systemic inflammation, which, in turn, may activate the HPA axis and alter neurotransmitter signalling [34,35,36]. Consequently, dysbiosis has been linked to an increased risk of anxiety, depression, and stress-related disorders [37].

## 3. GABA: Functional Role in the Central and Enteric Nervous Systems

**Figure 2:**
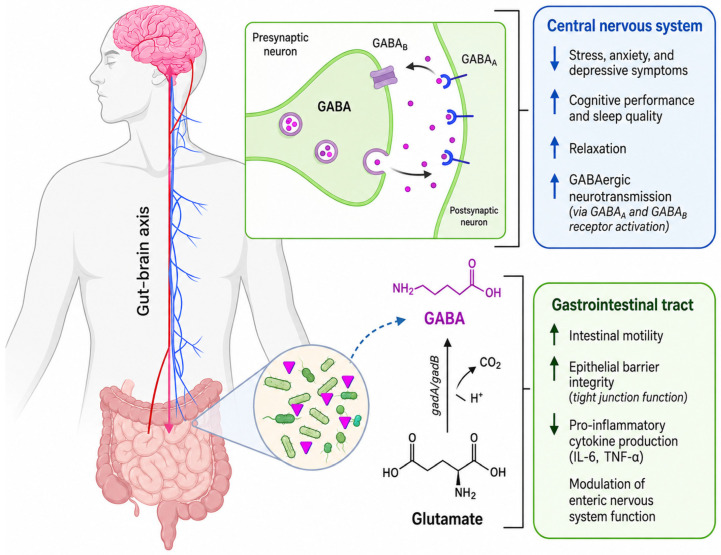
GABA as a key mediator in the gut–brain axis (created in BioRender (2026) https://BioRender.com/o4ukcvn (accessed on 26 May 2026)).

GABA plays a crucial role in both the central nervous system (CNS) and the enteric nervous system (ENS), modulating neuronal activity and gastrointestinal function [38] (Figure 2). In the CNS, GABA regulates mood, cognition, and stress responses, while in the ENS, it influences motility, secretion, and gut barrier integrity [38,39]. GABA is produced by enteric neurons and by the gut microbiota, including genera such as *Lactobacillus* and *Bifidobacterium*, and contributes to signalling within the gut–brain axis. Due to these features, GABA has been linked to the pathophysiology of both neurological and psychiatric disorders, including anxiety, depression, Parkinson’s and Alzheimer’s diseases, and functional gastrointestinal disorders such as irritable bowel syndrome (IBS) [40,41]. This section reviews the mechanisms behind GABA signalling and its functional importance in both the CNS and ENS.

### 3.1. GABA in the Central Nervous System (CNS)

GABA is the primary inhibitory neurotransmitter in the mammalian brain, vital for maintaining the excitatory–inhibitory balance and ensuring the stability of neuronal network activity [42,43,44]. In the CNS, its actions are mediated through three receptor subtypes: the ionotropic GABA*_A_* and GABA*_C_* receptors, and the metabotropic GABA*_B_* receptors [45]. GABA*_A_* and GABA*_C_* receptors regulate chloride ion influx, producing rapid synaptic inhibition. Conversely, GABA*_B_* receptors generate slower but longer-lasting effects by modulating potassium and calcium channels via Gi/o protein signalling [46]. In the brain, GABA is synthesised in GABAergic neurons through the decarboxylation of glutamic acid, a reaction catalysed by glutamate decarboxylase (GAD) with pyridoxal phosphate (PLP) as a cofactor [47]. In mammals, two GAD isoforms are present: GAD65 and GAD67, encoded by the *gad2* and *gad1* genes, respectively [12]. GAD65 is mainly localised at synaptic terminals, supporting rapid GABA synthesis in response to increased neuronal activity [48]. In contrast, GAD67 is distributed within the cytoplasm and maintains basal GABA production, ensuring continuous inhibitory signalling [49,50]. Effective GABAergic signalling is vital for maintaining neuronal stability, synchronising brain oscillations, and regulating cognitive and emotional functions [51]. Dysfunction of GABA signalling contributes to increased excitability in brain regions such as the prefrontal cortex, hippocampus, and amygdala, which are critical for mood regulation, memory processing, and stress responses [52,53]. Clinically, impaired GABAergic activity has been linked to the development of anxiety and depressive disorders, heightened stress reactivity, and, in severe cases, an increased risk of epileptic seizures [54,55,56,57].

### 3.2. GABA in the Enteric Nervous System (ENS)

The ENS, often called the “second brain,” is a vast network of over 500 million neurons and glial cells distributed throughout the gastrointestinal tract [58]. As a vital part of the gut–brain axis, the ENS regulates sensory, motor, and interneuronal functions, supporting gut homeostasis and overall health [59]. GABA is a key neurotransmitter that controls the activity of the enteric nervous system [60]. In the gut, GABA primarily acts via ionotropic GABA*_A_* and GABA*_C_* receptors, which are located on enteric neurons, epithelial cells, immune cells, and enteroendocrine cells (EECs) [61,62,63]. These receptors enable GABAergic signalling that influences intestinal motility, secretion, barrier integrity, and visceral sensitivity, thereby promoting gastrointestinal stability. GABA in the gut originates from various sources. Endogenous GABA is produced by GABAergic neurons through glutamate decarboxylase (GAD), which converts L-glutamate into GABA [38,63]. Apart from neurons, some EECs, including enterochromaffin (EC) cells, also express GABA*_A_* receptors and respond to GABA. Certain enteroendocrine-like cells produce GAD and may contribute to local GABA synthesis by interacting with enterochromaffin cells and other EECs [64,65]. External sources of GABA include the gut microbiota, particularly LAB and *Bifidobacterium*, which produce GABA via the GAD system [66,67]. Furthermore, consuming GABA-rich fermented foods can further increase intestinal GABA levels [68].

In the gut, GABA exerts both local and systemic effects. Within the ENS, GABAergic neurons are distributed throughout the myenteric and submucosal plexuses, where they regulate both excitatory cholinergic and inhibitory nitrergic pathways [69]. GABA released from these neurons modulates intestinal motility by inhibiting excitatory neurotransmission, thereby controlling smooth muscle contraction and peristalsis [61,63]. Moreover, GABAergic interneurons in the ENS participate in local reflex circuits that coordinate secretion and blood flow, ensuring optimal digestive function. Additionally, locally produced GABA may act in an autocrine or paracrine manner to modulate hormone release (e.g., serotonin, somatostatin) [70] and influence immune and epithelial cell functions, thereby maintaining gut barrier integrity [71].

Systemically, gut-derived GABA does not readily cross the blood–brain barrier; therefore, its effects on the central nervous system are believed to occur predominantly via indirect pathways involving neural, immune, and metabolic signalling [63,66,72]. Among these mechanisms, immune–brain communication is currently one of the strongest translational links between probiotic exposure and improvements in depressive symptoms, as supported by clinical evidence showing modulation of inflammatory markers following psychobiotic supplementation [73]. Vagal signalling is also considered an important pathway, particularly in preclinical models, in which microbiota-derived neuroactive compounds have been shown to influence vagal afferent activity and stress responsiveness. Through these interconnected pathways, microbial GABA may indirectly modulate neuroendocrine signalling, immune responses, mood regulation, cognitive function, stress reactivity, and hypothalamic–pituitary–adrenal (HPA) axis activity [70,74].

## 4. GABA-Producing Bacterial Strains

There is a growing body of research dedicated to identifying and screening GABA-producing bacterial strains, particularly from fermented foods, and investigating their presence in the human gut microbiota [75,76,77]. These studies help identify bacterial strains with high GABA-producing potential, which may have applications in probiotic and nutritional interventions to alleviate anxiety and depression.

### 4.1. Bacterial GAD System and GABA Biosynthesis Ability

Bacterial biosynthesis of GABA primarily occurs through the glutamate decarboxylase (GAD) system, which consists of two components: the glutamate decarboxylase enzyme (encoded by *gadA* or *gadB*) and the glutamate/GABA antiporter (encoded by *gadC*) [15,78]. In this process, L-glutamate is transported into the bacterial cell by the GadC antiporter and is decarboxylated by GAD in the presence of the cofactor pyridoxal-5′-phosphate (PLP) [79]. This reaction produces GABA and CO_2_ [80]. The newly formed intracellular GABA is then exported from the cell via the GadC antiporter, which also imports extracellular glutamate. This substrate–product exchange helps maintain the overall efficiency and balance of the GAD system [81]. GABA biosynthesis requires the conversion of intracellular protons. Therefore, the GABA operon is essential for the acid resistance of LAB [82]. The organization of the *gad* operon varies among bacterial species.

The presence of *gad* genes has been reported across multiple bacterial phyla, including Bacteroidetes (e.g., *Bacteroides*, *Parabacteroides*, *Alistipes*, *Odoribacter*, and *Prevotella*), Proteobacteria (e.g., *Escherichia*), Firmicutes (e.g., *Enterococcus*), and Actinobacteria (e.g., *Bifidobacterium*) [15]. Among these taxa, LAB, particularly members of the genera *Lactobacillus* and *Lactococcus*, have been extensively studied for their ability to synthesise GABA and their potential psychobiotic effects [83,84]. In LAB, the genomic structure of the GAD system varies considerably among species and strains. Typically, *gadB*, which encodes the glutamate decarboxylase enzyme, is located within the same operon as *gadC*, which encodes the glutamate/GABA antiporter, allowing coordinated transcription of both genes under the same promoter (*gadCB*) [15]. This arrangement has been well described in *Levilactobacillus brevis* [85] and *Lactococcus lactis* [86]. Additionally, the system is positively regulated by GadR, a transcriptional activator encoded upstream of *gadR* and expressed from its promoter, thereby enhancing gadB expression and increasing GABA synthesis [87,88]. Within LAB, the amino acid sequence similarity between GadA and GadB is relatively low [78]. However, their PLP-binding domains remain highly conserved, thereby maintaining catalytic function and enabling efficient glutamate decarboxylation [89,90]. In many LAB, *gadA* or *gadB* is present, including a high GABA-producing *L. brevis* strain [91,92,93,94]. Some *Lactiplantibacillus plantarum* strains harbour *gad* genes that are not organized into a classic operon because they are located on the plasmid [95]. In *Bifidobacterium* species, the GAD system, comprising the enzyme GadB and the antiporter GadC, functions synergistically to catalyse the conversion of glutamate to GABA. This GAD system has been well documented in *Bifidobacterium dentium*, *Bifidobacterium angulatum*, and *Bifidobacterium adolescentis* [15].

**Figure 3:**
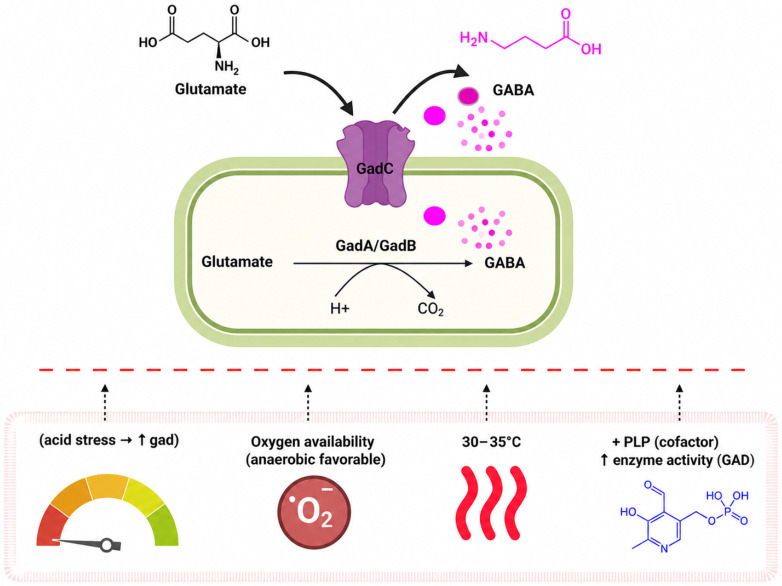
Environmental factors influencing bacterial GABA biosynthesis (created in BioRender. (2026) https://BioRender.com/7e56ziq (accessed on 26 May 2026)).

GABA production is highly strain-dependent and varies significantly even within the same bacterial species [15]. The efficiency of bacterial GABA biosynthesis is influenced by various environmental factors, including pH, temperature, oxygen availability, and substrate concentration (Figure 3). For instance, the growth of *L. brevis* NCL912 increased with temperature, peaking at 35 °C and declining at higher temperatures [96]. Similarly, *L. plantarum* DSM19463 exhibited its highest GABA synthesis rate (59 μM/h) within the temperature range of 30–35 °C [97]. Acidic conditions (pH = 5.4–5.5) and the availability of L-glutamate and glutamine as primary and secondary precursors are also key factors in bacterial GABA production [78]. Low pH induces expression of the GAD system, enabling bacteria to adapt to acid stress by increasing GABA production, though the optimal pH for maximal GABA synthesis varies by species. Di Cango et al. [97] showed that *L. plantarum* DSM19463 produced the maximum GABA (59 μM/h) at pH 6.0. However, Kumar et al. [98] observed that *Lacticaseibacillus paracasei* NFRI 7415 increased GABA production at 210 mM and pH 5.0. Additionally, GABA production by *Streptococcus thermophilus* Y2 was notably enhanced when the culture pH was periodically adjusted to 4.5 at 12-h intervals using NaOH or HCl after an initial 24 h of incubation [90]. Furthermore, supplementing culture media with glutamate has been shown to boost GABA biosynthesis. For example, cultivating *L. paracasei* NFRI 7415 in a medium containing 500 mM glutamate resulted in GABA production reaching 161 mM [99].

In vitro screening of bacterial GABA production is typically performed using de Man, Rogosa, and Sharpe (MRS) medium with varying concentrations of monosodium glutamate (MSG). MSG is used as a substrate because it can be hydrolysed to L-glutamic acid. This approach enables evaluation of strain-specific differences in GABA production efficiency and helps identify optimal substrate levels to maximise neurotransmitter synthesis. Duranti et al. [100] reported that 79% of tested *B. adolescentis* strains could convert MSG to GABA. Similarly, the highest GABA production by *L. plantarum* FNCC 260 in culture medium (1226.5 mg/L; ~11.9 mM) was observed at 100 mM MSG, compared to 809.2 mg/L (~7.85 mM) in MRS after 60 h of cultivation [78]. Under conditions with 100 mM of MSG, *L. plantarum* CGMCC 1.2437T produced 721.35 mM of GABA, which is 7 times higher than when no MSG was added [90]. Transcriptomic analysis showed that MSG increased the expression of key enzymes involved in carbohydrate metabolism, fatty acid synthesis, and amino acid metabolism [90]. Additionally, PLP, a cofactor of glutamate decarboxylase, is often added to enhance enzymatic activity and GABA production. Yang et al. [101] demonstrated that adding 0.2 mM and 0.02 mM PLP to the medium significantly enhanced GABA synthesis by *S. thermophilus* Y2. Meanwhile, Yogeswara et al. [102] reported that supplementing the medium with 0.6 mM PLP and 0.1 mM pyridoxine in *L. plantarum* FNCC 260 increased GABA output to 945. 3 mg/L (~9.17 mM) and 969. 5 mg/L (~9.40 mM), respectively. It was also shown that PLP at 10 or 100 µM in the culture medium significantly enhanced GABA biosynthesis by *L. paracasei* [99]. Furthermore, Cai et al. [103] demonstrated that adding 3% MSG and 2 mmol/L PLP to MRS medium markedly increased GABA production by *L. plantarum* FRT 7, reaching 1158. 6 mg/L (~11.2 mM). In contrast, cultivation of the same strain in standard MRS medium resulted in a considerably lower GABA concentration of 100. 75 mg/L (~0.98 mM).

### 4.2. GABA-Producing Bacterial Strains Isolated from Gut Microbiota

An initial screening of the Integrated Microbial Genomes/Human Microbiome Project database by Pokusaeva et al. [104] identified GAD orthologs in 26 bacterial genera. Among *Bacteroides*, the most abundant and dominant genus in the human gut microbiota, it was reported to be the most prevalent genus harbouring the *gadB* gene, followed by *Escherichia*, *Fusobacterium*, *Bifidobacterium*, and *Lactobacillus*. These findings were supported by a meta-analysis of 1159 gut bacterial genomes representing 919 species, which confirmed the widespread presence of *gadB* orthologs within *Bacteroides* [105]. In total, 45 *Bacteroides* strains harbouring *gadB* were identified, and GABA production was experimentally confirmed in selected strains, including *Bacteroides caccae* KLE1911, *Bacteroides vulgatus* KLE1910, *Bacteroides ovatus* KLE1170, *Bacteroides dorei* KLE1912, *Bacteroides uniformis* KLE1913, and *Bacteroides fragilis* KLE1758 [105]. Further studies demonstrated that nearly 96% of *Bacteroides* genomes derived from human intestinal isolates contain a complete GAD system [105]. Experimental analysis of 16 human gut-derived *Bacteroides* strains confirmed GABA production in the range of 0.09–60.84 mM, with the highest levels observed in *Bacteroides faecis* PB–SESWS, *Bacteroides fragilis* PB-SZSJC, *Bacteroides ovatus* DSM 1896, and *Bacteroides xylanisolvens* DSM 18836. In addition to *Bacteroides*, GABA-producing capacity has also been reported in *Parabacteroides* and *Eubacterium* species [106].

Members of the genus *Bifidobacterium* are also well-documented GABA producers. Barrett et al. [107] demonstrated that strains such as *B. dentium* DPC6333, NCFB2243, *Bifidobacterium infantis* UCC35624, and *B. adolescentis* DPC6044, isolated from the human gastrointestinal tract and faeces, reported concentrations ranging from 2.2 to 12.48 mg/mL (~21.3–121.0 mM). Subsequent studies confirmed GABA production in additional species, including *B. adolescentis*, *B. angulatum*, and *B. dentium* isolated from human faecal samples, with reported concentrations of 4.7–58.2 mM, 25.4–33.6 mM, and 23.9 mM, respectively [15]. Genomic analyses further supported these findings. An in silico study of 1022 *Bifidobacterium* genomes isolated from the human gastrointestinal tract revealed a high prevalence of *gad* genes, particularly in *B. adolescentis*, suggesting that this species may be a key contributor to GABA production in the human gut microbiota [100]. In vitro screening identified high-producing *B. adolescentis* strains, such as *B. adolescentis* PRL2019 and HD17T2H, that produced 7.6 mM and 9.43 mM GABA, respectively. Moreover, in vivo studies demonstrated that supplementation with *B. adolescentis* increased GABA levels in rat faecal samples [100]. Similarly, screening of 16 commensal intestinal isolates obtained from healthy human faecal samples identified *B. dentium* ATCC 27678 as an efficient GABA-producing strain capable of converting glutamate to GABA via the GadB-mediated decarboxylation pathway [104].

In *Lactobacillus*, a high GABA-producing potential has been observed in *L. plantarum* and *L. brevis* [15,66,77,78,87,99]. For instance, *L. brevis* DPC6108, isolated from infant faeces, demonstrated a strong ability to convert monosodium glutamate (MSG) into GABA, producing 11.01 and 20.47 mg/mL (~106.8 mM and ~198.5 mM) GABA from 10 and 20 mg/mL MSG, respectively, in MRS medium in vitro. Additional studies showed that supplementation with this strain significantly increased GABA levels during faecal fermentation, reaching 66.25 µg/mL (~0.642 mM) after 4 h and peaking at 70.72 µg/mL (~0.686 mM) after 9 h, suggesting its interaction with gut microbial communities [107]. Other strains, such as *L. plantarum* 90sk and *L. brevis* 15f, isolated from adult faeces, have also been identified as effective producers of GABA [108]. The amount of GABA produced by *L. plantarum* 90sk in culture medium was detected at 200 mg/L (~1.94 mM) [108]. Beyond GABA production, some strains exhibit additional beneficial properties, including antibiotic resistance and antioxidant properties [109]. More recently, *Limosilactobacillus fermentum* L18, isolated from faecal samples of healthy children, was shown to produce 62.37 mg/mL (~605 mM) of GABA under in vitro conditions after 24 h of cultivation [110]. *Lactococcus garvieae* MJF010, isolated from healthy human faeces, has also been identified as a strain with strong GABA biosynthesis capacity [111]. GABA-producing bacterial strains isolated from the human gut microbiota are summarized in Table 1.

**Table 1:** GABA-producing bacterial strains isolated from faecal human samples.

Bacterial Strain	Characterization	References
*Lactiplantibacillus plantarum* 299v (DSM 9843)	Isolated from healthy intestinal mucosa; GABA production: 5.81 mM (pH 5.7, 24 h incubation in MRS supplemented with 1% MSG)	[112]
*Lactiplantibacillus plantarum* 90sk	Isolated from the human gastrointestinal tract; GABA production: 200 mg/L (~1.94 mM) in MRS supplemented with 1% MSG; increased to 843 mg/L (~8.18 mM) upon PLP addition in the late stationary phase; exhibits antibiotic resistance and antioxidant activity.	[108]
*Levilactobacillus brevis* DPC6108	Isolated from infant faeces; convert 100% MSG (10–20 mg/mL) to 11.03 and 20.47 mg/mL GABA (~107.0 mM and ~198.5 mM); higher MSG concentrations (30–50 mg/mL) reduce conversion to 64.6–94.4%, yielding 28.02–32.32 mg/mL GABA (~271.8–313.5 mM); increased GABA production observed in fermented faecal slurry	[112]
*Bifidobacterium adolescentis* PRL2019	Isolated from the human intestine; in vivo GABA production: 7.06 mM; MSG-to-GABA conversion: 64.97% after 24 h incubation.	[100]
*Bifidobacterium adolescetis* HD17T2H	Isolated from human faeces; in vivo GABA production: 9.43 mM; MSG-to-GABA conversion: 86.80% after 24 h incubation.	[100]
*Bifidobacterium adolescentis* DPC6044 *Bifidobacterium dentium* DPC6333 *Bifidobacterium infantis* UCC3562	Isolated from infant faeces; MSG-to-GABA conversion ranged from 22–60.9% (2.2–6.09 mg/mL; ~21.3–59.1 mM) at 10 mg/mL MSG and 15.9–61.6% (3.17–12.32 mg/mL; ~30.7–119.5 mM) at 20 mg/mL MSG	[107]
*Bifidobacterium adolescentis* 150	Isolated from the human gastrointestinal tract; GABA production: 5.6 g/L (~54.3 mM).	[15]
*Bacteroides faecis* PB-SESWS *Bacteroides fragilis* PB-SZSJC *Bacteroides ovatus* DSM 1896 *Bacteroides xylanisolvens* DSM 18836	Isolated from human faeces; GABA production ranged from 46.59 to 60.84 mM after 48 h incubation in mY-CFA-Glu medium.	[106]
*Lactococcus garvieae* MJF010	Isolated from human faeces; high GABA-producing activity at 35 °C and pH 5; PLP did not affect GAD activity	[111]

### 4.3. GABA-Producing Bacterial Strains Isolated from Food Products

Numerous studies have shown that LAB can synthesise GABA in food matrices, including fermented vegetables, fermented dairy products, and cheeses [66,113]. Therefore, recent research has concentrated on the isolation, characterisation, and technological evaluation of food-derived GABA-producing strains (Table 2). The aim is to develop functional foods naturally enriched with GABA and to improve the health-promoting properties of fermented products [114].

High GABA-producing strains, including *L. paracasei* PF6, *Lactobacillus delbrueckii* subsp. *bulgaricus* PR1, *L. lactis* PU1, and *L. brevis* PM17, have been identified in cheeses such as Pecorino di Filiano and Pecorino del Reatino [115]. The highest GABA concentrations were recorded in Pecorino Marchigiano (289 mg/kg), Pecorino del Reatino (290 mg/kg), Pecorino Leccese (290 mg/kg), Pecorino Umbro (330 mg/kg), and Pecorino di Filiano (391 mg/kg). Among the strains tested, *L. paracasei* PF6, *L. delbrueckii* subsp. *bulgaricus* PR1, *L. lactis* PU1, *L. plantarum* C48, and *L. brevis* PM17 showed the highest GABA production levels (99.9, 63.0, 36.0, 16.0, and 15.0 mg/kg, respectively) [115]. High GABA-producing LAB strains have also been isolated from plant-based fermented foods, including Korean kimchi, traditional Chinese paocai, and Japanese fermented fish. For example, *L. plantarum* FRT7, isolated from paocai, produced 1158.6 mg/L (~11.2 mM) of GABA under optimised conditions [103].

Recent studies highlight *L. brevis* as one of the most efficient LAB species for GABA production [116,117]; however, its capacity is highly strain-dependent and affected by the source of isolation and fermentation conditions [86,118,119]. For example, *L. brevis* NCL912, isolated from paocai, produced approximately 15.4 g/L (~149 mM) of GABA under optimised conditions [103], while *L. brevis* K203, isolated from kimchi, produced 44.4 g/L (~431 mM) [120]. Similarly, Lim et al. [121] reported that *L. brevis* HYE1, also isolated from kimchi, produced 18.76 mM GABA under optimal in vitro conditions, while *L. brevis* CRL1942, isolated from quinoa sourdough, produced up to 255 mM GABA in the presence of 4.5% MSG [122]. Other high-producing strains include *L. brevis* CRAI, isolated from organic tomatoes, which produced 179.15 mM GABA from an initial concentration of 236.53 mM (4%) MSG [123]. Furthermore, co-cultivation strategies, such as those of *L. delbrueckii* subsp. *bulgaricus* with *S. thermophilus* IFO13957 [124], or *L. brevis* with *L. plantarum* from Egyptian dairy products [125], have been shown to enhance GABA production. In addition, strains isolated from Mexican milk kefir grains, including *L. lactis* (BIOTEC006-008) and *Leuconostoc pseudomesenteroides* (BIOTEC012), have been identified as GABA producers with potential probiotic properties [126]. Similarly, six *L. lactis* strains, namely LEY6, LEY7, LEY8, LEY11, LEY12, and LEY13, isolated from raw camel milk, produced GABA at concentrations ranging from 1.74 mM to 1.80 mM [127]. Moreover, all strains produced GABA in a Cabrales-like mini-cheese model, with GABA accumulation ranging from 350 mg/kg (LEY6) to 457 mg/kg (LEY12).

It should also be noted that the high GABA concentrations reported under optimised in vitro culture conditions may not accurately reflect the physiological levels achieved within the gastrointestinal tract in vivo. In the intestinal environment, factors such as substrate availability, microbial competition, intestinal absorption, and host metabolism may substantially influence microbial GABA production, bioavailability, and overall biological activity. Nonetheless, in vitro screening provides valuable preliminary insight into the GABA-producing capacity of psychobiotic strains and remains an important step in identifying candidate microorganisms for further translational and clinical investigation, including their potential incorporation into functional foods and into microbiota-based interventions to support individuals affected by stress-related or neuropsychiatric disorders.

**Table 2:** GABA-producing bacterial strains isolated from a wide range of fermented foods.

Microorganism	Source	GABA Production	References
*Levilactobacillus brevis* CECT 8183/CECT 8181/CECT 8182 *Lactococcus lactis* CECT 8184	Goat cheese; sheep cheese; goat cheese; goat cheese	0.96, 0.94, 0.99, 0.93 mM, respectively (in a wheat flour solution after 24 h incubation)	[128]
*Lactiplantibacillus plantarum* FNCC 260	Fermented cassava	1226.5 mg/L (~11.9 mM) (in MRS supplemented with 100 mM MSG after 60 h of cultivation)	[102]
*Lactiplantibacillus plantarum* DRBA1/DVBA1 *Lactococcus lactis* IBA *Levilactobacillus brevis* CRAR *Levilactobacillus brevis* CRAI	Organic tomatoes (cherry red, plum, grape green, grape red)	2.91, 2.25, 36.21, 165.24, and 179.15 mM, respectively (in MRS with 4% MSG after 48 h of cultivation)	[123]
*Lentilactobacillus buchneri* MS *Levilactobacillus brevis* K203/L-32/HY1/877 G*Lactococcus lactis* subsp. *lactis* B	Kimchi, yoghurt	251, 430.57, 349.1, 18.76, 18.94, 62.16 mM, respectively; cultivation conditions: MRS with 5% MSG (38 h, MS); MRS with 6% glutamate (72 h, K2023); MRS with 118 mM MSG (58 h, L-32); MRS with 2.38% MSG (48 h, HY1); MRS with 59.13 mM/L MSG (15 h, 877 G); GM17 with 5mM MSG (5 days, *lactis* B)	[120,121,123,127,129,130]
*Lacticaseibacillus paracasei* 15 C *Lacticaseibacillus rhamnosus* 21D-B *Streptococcus thermophilus* 84 C	Raw milk cheese	14.8, 11.3, 80 mg/kg, respectively (in M17 for 15C) and MRS for 21D-B and 84C, supplemented with 7.0 mM L-glutamate, after 24 h of incubation)	[131]
*Lacticaseibacillus paracasei* NFRI 7415	Japanese fermented fish (funazushi)	161 mM (after 144 h of cultivation)	[99]
*Levilactobacillus brevis* NCL912	Chinese paocai	149 mM (in MRS supplemented with 3% MSG after 48 h of cultivation)	[132]
*Levilactobacillus brevis* MG5552/MG5405/MG5261/MG5522	Fermented food (Republic of Korea)	0.624, 0.585, 0.591, 0.979 mg/mL (~6.05 mM, ~5.67 mM, ~5.73 mM, ~9.50 mM), respectively (in MRS supplemented with 1% MSG)	[133]
*Lactiplantibacillus plantarum* subsp. *plantarum* LSI2-1	Thai fermented food	22.94 g/L (~222.5 mM) (in GYP broth with 3% MSG after 72 h of cultivation)	[134]
*Lactiplantibacillus plantarum* 45a/44d	Cambodian fermented foods (paork kampeus)	20.34 and 16.47 mM (in MRS supplemented with 2% MSG after 48 h of cultivation)	[135]
*Companilactobacillus futsaii* CS3	Thai fermented shrimp (kung-som)	242.44 mM (in MRS supplemented with 1% MSG after 72 h of cultivation)	[136]
*Lactococcus lactis* BIOTEC008	Mexican milk kefir grain	0.29 mM (in MRS supplemented with 5 mM MSG after 48 h of cultivation)	[126]
*Lactococcus lactis* LEY6/LEY7/LEY12/LEY13	Raw camel milk	1.74, 1.8, 1.32 and 1.22 mM, respectively (in medium supplemented with 5 mM MSG after 5 days of cultivation)	[127]
*Lentilactobacillus buchneri* WPZ001	Chinese fermented sausage	1250.97 mM (in MRS supplemented with 100 g/L MSG and 30 g/L xylose after 96 h of cultivation)	[137]
*Latilactobacillus curvatus* N-19 *Furfurilactobacillus rossiae* ED-1 *Lactiplantibacillus plantarum* ED-10 *Levilactobacillus brevis* E-25	Sourdough	14.17, 11.04, 15.47 and 11.92 mM, respectively (in MRS supplemented with 53 mM MSG after 96 h of cultivation)	[138]
*Lactococcus lactis*	Yam pickles	10.7 mM (in MRS supplemented with 5% MSG after 48 h of cultivation	[139]

## 5. Mechanisms of Psychobiotic Action in the Microbiota–Gut–Brain Axis

The increasing interest in identifying microorganisms capable of producing neuroactive compounds, especially GABA, has led to the development of the concept of psychobiotics [16]. Psychobiotics are a class of probiotics with potential importance for the prevention and treatment of psychiatric disorders [7,16,30,78,83]. Much attention has been given to LAB, as selected strains are classified as Generally Recognised as Safe (GRAS) by the United States Food and Drug Administration (FDA) and have Qualified Presumption of Safety (QPS) status from the European Food Safety Authority (EFSA) (Agriopoulou et al.) [140]. LAB often display several probiotic properties, including tolerance to gastric acidity and bile salts [141], adherence to the intestinal epithelium [142], and antagonistic activity against pathogenic microorganisms [143]. These properties, together with LAB’s ability to modulate the composition and metabolic activity of the gut microbiota in ways that influence central nervous system function, make them promising candidates for psychobiotics.

### 5.1. Microbial Metabolites and Signalling Pathways in the Microbiota–Gut–Brain Axis

**Figure 4:**
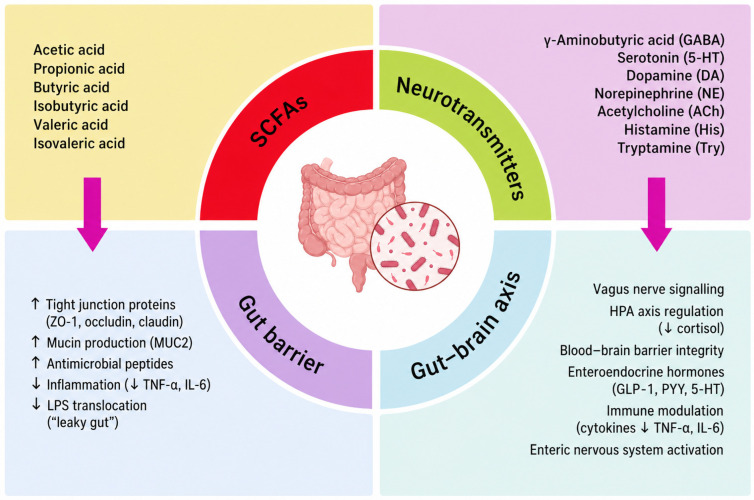
Microbial metabolites and neurotransmitters mediating communication along the microbiota–gut–brain (MGB) axis and contributing to intestinal barrier regulation (created in BioRender (2026) https://BioRender.com/p64hs1u (accessed on 26 May 2026)).

Although this review primarily focuses on the role of GABA in regulating the MGB axis, this pathway is considered within a broader network of microbial-derived signals, including SCFAs and tryptophan metabolites, which collectively orchestrate host–microbiota communication, regulate gut barrier integrity, and influence CNS function. Psychobiotics may affect cognitive function through complex, bidirectional interactions within the MGB axis [27,58,68,72,105,144]. These effects are mediated by dynamic molecular crosstalk at the interface between the intestinal microbiota and the host [3,7,17,18,145]. Current evidence indicates that these interactions are driven by multiple interconnected mechanisms, with the production of bioactive metabolites playing a central role [146,147]. These molecules act as signalling mediators that can influence the CNS both directly and indirectly via peripheral pathways, including immune, endocrine, and neural signalling routes [148,149] (Figure 4).

#### 5.1.1. Role of SCFAs in the Microbiota–Gut–Brain (MGB) Axis

Microbiota-derived SCFAs are vital signalling molecules involved in communication between the gut microbiota and the host. These compounds are mainly produced by bacterial fermentation of dietary fibres and other non-digestible substrates that reach the colon [147,148,149]. The primary SCFAs—acetate (C2), propionate (C3), and butyrate (C4)—can attain high millimolar concentrations in the human colon [150]. SCFAs play a crucial role in maintaining intestinal barrier integrity, regulating host metabolism, and modulating immune tolerance and function [149]. Among them, butyrate is particularly notable for its ability to influence nervous system function, including modulation of GABAergic neuronal activity [148]. SCFAs are absorbed by intestinal cells via multiple transport mechanisms, including passive diffusion and carrier-mediated transport. Their dissociated forms are transported by monocarboxylate transporters such as MCT1 (SLC16A1) and MCT4 (SLC16A3), as well as sodium-coupled monocarboxylate transporters, including SMCT1 (SLC5A8) and SMCT2 (SLC5A12) [151,152]. Once absorbed, most SCFAs are metabolised by colonocytes, with butyrate serving as their primary energy source [150].

The effects of SCFAs on the gut–brain axis are mediated by multiple signalling pathways that link intestinal microbial activity to neural and behavioural outcomes [149,150]. SCFAs activate G-protein-coupled receptors, such as FFAR2 and FFAR3, expressed on enteroendocrine L-cells, promoting the release of hormones including glucagon-like peptide-1 (GLP-1) and peptide YY (PYY), thereby influencing vagal afferent signalling to the brain [147,148]. Furthermore, SCFAs can enter the systemic circulation and affect CNS function by modulating blood–brain barrier integrity, neuroinflammation, and microglial activation [152]. At the molecular level, SCFAs, especially butyrate, act as epigenetic regulators by inhibiting histone deacetylases (HDACs), thereby promoting histone acetylation and altering gene expression linked to neurotransmission and synaptic plasticity [153]. In a preclinical mouse model of metabolic dysfunction, sodium butyrate increased plasma and hippocampal GABA levels and upregulated the GABA-synthesising enzyme glutamic acid decarboxylase (GAD67), an effect associated with enhanced histone acetylation at the GAD67 promoter [154]. Beyond its role as an epigenetic regulator, butyrate plays a significant part in regulating intestinal immune responses. It has been shown to activate GPR109A, thus promoting the differentiation of regulatory T cells (Tregs), while simultaneously suppressing pro-inflammatory gene expression by inhibiting the TLR4/NF-κB signalling pathway [149,155]. Thananimit et al. [156] demonstrated that *L. paracasei* SD1 and *L. rhamnosus* SD11 produce detectable butyrate concentrations (250–360 mg/L), considerably exceeding levels observed in *L. plantarum* O4T10E (2.89 mg/L). Conversely, in a mouse model of Parkinson’s disease, propionate restored striatal GABA levels, thereby re-establishing excitatory–inhibitory balance and improving motor function [150,157]. Alterations in SCFA profiles have been linked to neuroimmune and neuropsychiatric conditions, including depression, anxiety, and autism spectrum disorders [158].

#### 5.1.2. Modulation of Neurotransmitter Biosynthesis and Signalling in the Microbiota–Gut–Brain (MGB) Axis

The gut–brain axis is strongly regulated by microbiota-derived metabolites, including GABA, SCFAs, tryptophan-derived metabolites, and bile acid derivatives [16,27,141,159]. These metabolites do not act in isolation but interact within a complex metabolic and signalling network, collectively influencing intestinal barrier integrity, neuroimmune homeostasis, neurotransmitter biosynthesis, and host–brain communication. Among these metabolites, tryptophan-derived compounds, particularly indole and its derivatives, play an important role in MGB axis communication [159]. These compounds are generated through microbial tryptophan metabolism and influence host physiology through serotonergic, kynurenine, and aryl hydrocarbon receptor (AhR)-mediated signalling pathways [160]. Indole metabolites have been shown to regulate intestinal barrier integrity, modulate immune homeostasis, reduce neuroinflammatory responses, and influence enteroendocrine and vagal signalling, thereby indirectly affecting central nervous system function [159,161]. Similarly, bile acid derivatives participate in gut–brain axis communication through bile acid-sensitive receptors, including the farnesoid X receptor (FXR), which is highly expressed in intestinal epithelial cells, and the G protein-coupled bile acid receptor TGR5 (GPBAR1), present in enteroendocrine, immune, and enteric neuronal cells [161,162]. Through these receptors, bile acids contribute to metabolic regulation, intestinal barrier integrity, immune homeostasis, and neuroendocrine signalling [161].

In addition, evidence indicates that probiotic microorganisms can modulate serotonin (5-HT) biosynthesis. Myung et al. [163] investigated the effects of *L. plantarum* KBL396, isolated from a healthy Korean adult, on behavioural outcomes, 5-HT production, and immune modulation. In vitro experiments demonstrated significant upregulation of *TPH1* expression, and administration of the strain in a chronic social defeat stress mouse model attenuated depressive-like behaviours, increased serum 5-HT concentrations, and modulated immune cell populations [163]. Importantly, these preclinical observations were partially supported by a randomised, double-blind, placebo-controlled clinical trial (KBL396 group: *n* = 62; placebo group: *n* = 30), in which supplementation with *L. plantarum* KBL396 significantly increased serum levels of 5-HT and dopamine in human participants [163]. Similarly, Oh et al. [164] reported that fermented milk products containing *Lactobacillus rhamnosus* 4B15 enhanced peripheral 5-HT biosynthesis and influenced stress-related behaviours in mice. Zhang et al. [165] further demonstrated that *L. rhamnosus* LRa05 improved gastrointestinal motility in a constipation model by upregulating tryptophan hydroxylase 1 (*Tph1*) expression and increasing 5-HT levels, thereby activating 5-HT4 receptor-mediated signalling pathways.

Within this broader signalling network, GABA should not be viewed as an isolated mediator of MGB communication. Microbial GABA appears to exert predominantly indirect effects via enteric, vagal, immune, and metabolic pathways. For example, GABAergic signalling may influence serotonin secretion from enterochromaffin (EC) cells and modulate serotonergic neurotransmission along gut–brain axis pathways [162,165]. In the gut, microbial GABA acts locally via GABA*_A_* and GABA*_B_* receptors on EC cells, which may regulate or suppress the release of 5-HT in response to stimulation into the surrounding tissue [166]. However, compared with GABA, SCFAs, particularly butyrate, not only enhance serotonin biosynthesis by upregulating *Tph1* but also modulate serotonin transporter (*Sert*) expression [166].

Importantly, probiotic supplementation with psychobiotic strains may enhance these biological effects not only through the direct production of neuroactive compounds such as GABA, but also by modulating the composition and metabolic activity of the gut microbiota. Changes in microbial diversity, substrate utilisation, and cross-feeding interactions may collectively promote the production of bioactive metabolites (SCFAs, indole derivatives), and neurotransmitter-related compounds, including GABA, serotonin precursors, dopamine-related metabolites, and other neuroactive molecules involved in MGB axis communication [162,165]. These changes may strengthen intestinal homeostasis, improve neuroimmune regulation, and support host–brain communication [94,163,166]. Taken together, these findings suggest that psychobiotics may influence MGB axis communication not exclusively through GABAergic mechanisms, but through broader microbiota-mediated modulation of metabolic and neurochemical pathways that collectively contribute to central nervous system regulation and mental health outcomes.

#### 5.1.3. Regulation of Intestinal Barrier Integrity in the Microbiota–Gut–Brain (MGB) Axis

Alterations in mucosal permeability and disruption of epithelial barrier integrity are key mechanisms in the development of many gastrointestinal disorders, including inflammatory bowel disease (IBD) and IBS. Barrier impairment can promote systemic inflammation and disturb gut–brain axis signalling, which has been linked to neuropsychiatric symptoms such as depression [167,168,169]. In Alzheimer’s disease, barrier dysregulation may alter peripheral GABA levels, potentially impacting central GABAergic signalling via humoral and vagal pathways, emphasising the importance of an intact intestinal barrier for proper GABA function [42]. Probiotic strains of *Lactobacillus* and *Bifidobacterium* maintain epithelial barrier integrity through various mechanisms [170]. They stimulate mucin secretion and increase the expression of tight junction (TJ) proteins, including occluding (OCLN), claudins (CLDN), and zonula occludens-1 (ZO-1). Simultaneously, probiotics modulate immune responses by decreasing pro-inflammatory cytokines (IL-6, IFN-γ, TNF-α, IL-1β) and increasing anti-inflammatory mediators (IL-10, TGF-β), partly by inhibiting the NF-κB signalling pathway [166,171,172]. These effects collectively enhance barrier function and safeguard epithelial cells from apoptosis [110].

Several GABA-producing strains belonging to the genera *Lactobacillus* and *Bifidobacterium* have also been associated with improved intestinal barrier integrity and reduced intestinal inflammation, suggesting that maintenance of epithelial homeostasis may be an important indirect mechanism by which GABA-producing psychobiotics influence MGB axis communication [173,174,175,176,177]. By preserving barrier function and limiting systemic inflammation, these strains may help maintain proper neuroimmune and GABAergic signalling between the gut and the central nervous system. Protective effects on epithelial TJ integrity have been demonstrated in lipopolysaccharide (LPS)-induced and experimental colitis models following administration of several probiotic strains, including *Lactobacillus acidophilus* LA1 [173], *Limosilactobacillus reuteri* FN041 [174], *Lactobacillus helveticus* ASCC 511 [175], and *Ligilactobacillus salivarius* SMXD51 [176]. For example, Lépine et al. [177] demonstrated that *L. acidophilus* W37 enhances intestinal barrier function. This effect was mediated by the upregulation of TJ proteins, including OCLN, CLDN4, CLDN15, and CLDN16, in Caco-2 cells compromised by pathogenic *Salmonella typhimurium*. Similar results have been reported by Kainulainen et al. [178], who found that *L. acidophilus* LAB20 increased barrier integrity in Caco-2 cells and prevented LPS-induced IL-8 production in HT-29 cells. Additionally, Hummel et al. reported that *L. acidophilus*, *L. fermentum*, *Lactobacillus gasseri*, and *L. rhamnosus* increased transepithelial electrical resistance (TEER) in T84 intestinal epithelial monolayers by modulating E-cadherin and catenin expression. Another study indicated that *B. dentium* N8 significantly alleviated LPS-induced intestinal barrier injury by upregulating TJ proteins and reducing inflammation in a Caco-2 cell model [179].

#### 5.1.4. Regulation of Intestinal Inflammation in the Microbiota–Gut–Brain (MGB) Axis

Numerous animal studies have demonstrated the protective effects of *Lactobacillus* strains against colitis and their potential to reduce the risk of inflammatory bowel disease (IBD) [180,181,182]. In a murine colitis model, administration of *L. rhamnosus* MTCC-5897 significantly increased TJ protein expression [183]. Additionally, probiotic treatment reduced pro-inflammatory markers (IL-4, TNF-α, CRP, and MPO activity) and increased anti-inflammatory mediators such as TGF-β and intestinal IgA, suggesting a protective role against colitis-associated inflammation. Nazari et al. [184] reported that *L. reuteri* supplementation alleviated dextran sodium sulfate (DSS)-induced colitis in mice. This beneficial effect was linked to enhanced expression of ZO-1 and CLDN1, increased SCFA production, and restored intestinal barrier integrity, ultimately reducing intestinal inflammation by decreasing pro-inflammatory TNF-α, IL-1β, and IL-6 levels and increasing anti-inflammatory IL-10 levels. In another study*, L. reuteri* alleviates intestinal barrier damage in IPEC-1 cells caused by infection with the enterotoxigenic *Escherichia coli* K88 strain by preventing disruption of ZO-1 protein [185]. Shin et al. [186] found that *L. brevis* Bmb6 regulated the cross-talk between inflammatory mediators and TJ proteins in mice with DSS-induced colitis, thereby restoring gut epithelial structural integrity and alleviating colitis symptoms. Treatment with *L. brevis* Bmb6 significantly suppressed the expression of pro-inflammatory cytokines, including TNF-α and IFN-γ, in mice with DSS-induced colitis. Furthermore, multi-strain probiotic formulations, including combinations of *L. plantarum*, *L. reuteri*, *Bifidobacterium* spp., *L. lactis*, *L. fermentum*, and *S. thermophilus*, have been shown to maintain intestinal barrier integrity and reduce inflammatory response. In Caco-2/THP-1 co-culture and colitis mouse models, these formulations preserved occludin expression, activated the AMPK pathway, and reduced the production of pro-inflammatory mediators, including TNF-α, IL-1β, and IL-8, through NF-κB inhibition [187,188]. Similarly, numerous animal studies have demonstrated that members of the *Bifidobacterium* exert protective effects in experimental models of colitis [189,190]. Specific strains, including *Bifidobacterium longum* YS108R, 51A, BGN4, and LC67, alleviated DSS- or TNBS-induced colonic injury by strengthening mucosal barrier integrity and regulating pro-inflammatory signalling pathways, thereby reducing pro-inflammatory cytokines such as IL-6, IL-1β, and TNF-α [191,192]. Likewise, *Bifidobacterium infantis* ATCC 15697, *Bifidobacterium bifidum* BGN4-SK, and *Bifidobacterium lactis* A6 showed protective effects against DSS-induced colitis in mice, supporting the role of *Bifidobacterium* in maintaining intestinal homeostasis [193,194].

In summary, the integrity of the intestinal barrier and the production of microbial metabolites, including SCFAs and tryptophan-derived compounds, are crucial for microbiota–gut–brain communication. SCFAs, especially butyrate, regulate immune responses, enhance TJ protein expression, modulate histone acetylation, and influence neurotransmitter systems, including GABAergic and serotonergic signalling. Probiotic strains of *Lactobacillus* and *Bifidobacterium* have been shown to maintain barrier integrity, reduce pro-inflammatory cytokines, and increase the production of bioactive metabolites, collectively supporting gut homeostasis and influencing neurobehavioural outcomes. Among these metabolites, GABA has attracted attention as a key inhibitory neurotransmitter, with bacteria capable of producing GABA emerging as potential psychobiotics. Changes in intestinal barrier function can impact peripheral GABA levels. They may influence central GABAergic signalling through humoral and vagal pathways, emphasising the importance of preserving barrier integrity for proper GABA function.

## 6. Beneficial Effects of GABA-Producing Psychobiotics in Mental Health

Neuropsychiatric disorders, including depression, anxiety, mood disturbances, and cognitive impairment, are increasingly linked to changes in gut microbiota composition and dysregulation of the MGB axis [195]. Growing evidence indicates that gut microbiota dysbiosis can disrupt this bidirectional communication system, thereby affecting neuroinflammatory pathways, neurotransmitter metabolism, and stress responses in the host, contributing to the development of mental health disorders [196,197,198]. Notably, the prevalence of mood disorders, anxiety, and cognitive dysfunction has been rising among young populations. This trend is partly connected to dietary patterns typical of the Western diet, which is often high in saturated fats, refined sugars, and heavily processed foods. Such a pro-inflammatory diet may foster systemic inflammation, gut microbiota imbalance, and disturbances in the MGB axis, increasing susceptibility to mood disorders, including depression [199]. Consequently, more attention is being directed towards microbiota-targeted therapies aimed at restoring gut microbial balance and improving mental health outcomes.

**Figure 5:**
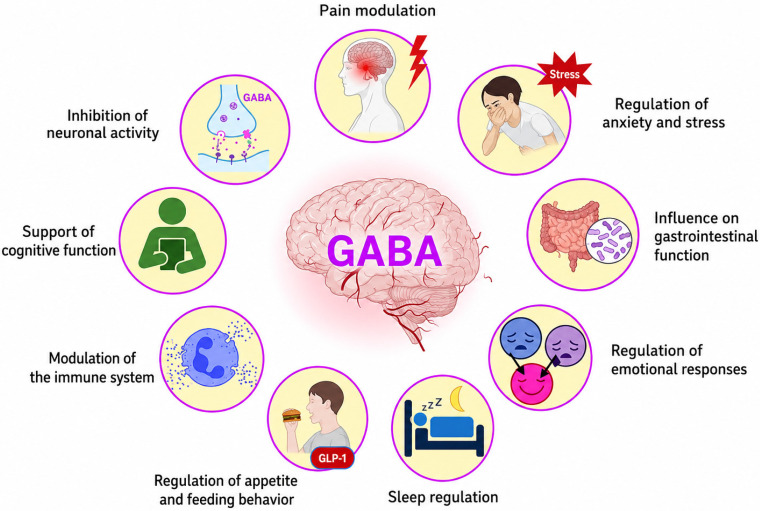
Biological functions of GABA in neural and systemic regulation (created in BioRender (2026) https://BioRender.com/14r05fd (accessed on 26 May 2026)).

GABA is the primary inhibitory neurotransmitter in the CNS and plays a vital role in controlling neuronal excitability and maintaining neural homeostasis [200]. Beyond its central function in the brain, GABA influences several physiological processes, including modulation of pain perception, regulation of stress and anxiety responses, emotional regulation, cognitive function, sleep, appetite and feeding behaviour, gastrointestinal activity, and immune system performance [114] (Figure 5).

Numerous preclinical studies and clinical trials indicate that GABA-producing probiotic strains may alleviate symptoms of stress-related gut–brain disorders and influence resting brain activity, cognitive performance, and memory [201,202,203]. These strains have been shown to affect symptoms of stress-related gut–brain disorders and to regulate resting-state brain activity, cognitive performance, and memory through microbiota-targeted interventions [204,205,206].

### 6.1. Preclinical Studies on Animal Models

Preclinical studies using animal models have shown that supplementation with specific *Lactobacillus* and *Bifidobacterium* strains capable of producing GABA has beneficial effects on nervous system regulation, including modulation of stress responses and reductions in anxiety- and depression-like behaviours [207,208,209]. The psychobiotic potential of *L. rhamnosus* JB-1 has been studied in relation to GABAergic signalling and stress-related behaviours [209]. Oral administration of *L. rhamnosus* JB-1 (1 × 10^9^ CFU) for 28 days resulted in modulation of the GABAergic system, accompanied by decreased circulating corticosterone levels and reduced anxiety- and depressive-like behaviours. In another study, mice subjected to early-life stress showed modulated behavioural responses following administration of *L. plantarum* PS128, as evidenced by improved psychomotor function and reduced anxiety-like behaviours [210]. The mechanism involves increasing dopamine transporter and β-arrestin expression while reducing phosphorylation of dopamine and cAMP-regulated phosphoprotein (DARPP-32) [211]. In a similar preclinical study, Cowan et al. [212] showed that supplementation with *L. helveticus* R0052 and *L. rhamnosus* R0011 mitigated the adverse effects of early-life stress in infant rats. In particular, *L. helveticus* R0052 was reported to alleviate stress-induced impairments in neuroplasticity and neurogenesis associated with chronic stress exposure. These effects were accompanied by reduced HPA axis activity and autonomic nervous system activation, as reflected by decreased cortisol and catecholamine levels. Similarly, Lonstein et al. [213] demonstrated that *L. rhamnosus* HN001 reduced stress-associated maternal behaviours in rats, including postpartum anxiety-like behaviours. Decreased levels of norepinephrine, dopamine, and serotonin in the prefrontal cortex supported these effects. However, Luo et al. [214] reported that *L. helveticus* NS8 alleviated depressive- and anxiety-like behaviours in a rat model of HA-induced neurological dysfunction, likely through modulation of depression-related neuronal pathways. In another study, long-term supplementation with *L. paracasei* K71 improved cognitive performance in mice, as demonstrated by enhanced performance in the Barnes maze and passive avoidance tests [215]. This effect was accompanied by an upregulation of brain-derived neurotrophic factor (BDNF) expression in the hippocampus. Similarly, *L. plantarum* GM11 has shown potential in alleviating depressive-like behaviours and other mental health disturbances in rat models. These effects may be associated with the modulation of monoamine neurotransmitter levels, normalisation of HPA axis activity, and regulation of the cAMP response element-binding protein (CREB)–BDNF signalling pathway [216]. On the other hand, evidence from animal studies supports the psychobiotic potential of specific *Bifidobacterium* strains. For instance, supplementation with *Bifidobacterium breve* CCFM1025 has been shown to alleviate anxiety- and depressive-like behaviours in a murine model. These effects were accompanied by modulation of the HPA axis and attenuation of stress-induced inflammatory responses [217]. Similar effects have been shown for *B. longum* Rosell^®^-175. The use of this strain in mice, in combination with *L. helveticus* R0052, influenced the HPA axis under chronic stress, alleviating its effects [218]. In addition, research conducted by Jarosz et al. [219] provided evidence that supplementation of mice’ diets with *L. rhamnosus* JB-1 and *B. longum Rosell*^®^-175 modifies the expression of brain proteins involved in metabolic and immune processes.

### 6.2. Clinical Human Studies on Psychobiotics

In humans, psychobiotics are increasingly investigated as a therapy for mental health disorders, emphasising the role of the gut microbiota in regulating psychological well-being through the gut–brain axis [220]. A meta-analysis by Zhang et al. [221], covering 13 studies, suggested that psychobiotic interventions targeting the gut microbiota could be a promising approach for managing mild-to-moderate depression. However, other meta-analyses show that the effects of probiotics on mental health outcomes are varied and may depend on the population studied and the parameters evaluated. For example, Ng et al. [222] found that probiotic supplementation provided statistically significant benefits for patients with mild to moderate depression. Conversely, Chao et al. [223] reported that the overall effect of probiotics on depressive symptoms in both depressed individuals and healthy adults was not statistically significant. Similarly, Gao et al. [224] noted variability in probiotic responses, showing different effects on anxiety and depression across studies. Nonetheless, many studies support the positive influence of psychobiotic supplementation on human mental health [225]. For instance, Akkasheh et al. [226] conducted a randomised, double-blind, placebo-controlled clinical trial involving 40 patients with depression, who were assigned to receive either a probiotic formulation (*L. acidophilus*, *L. casei*, *B. bifidum*) or a placebo for 8 weeks. The findings indicated that probiotic supplementation significantly lowered Beck Depression Inventory (BDI) scores compared to the placebo group. Similarly, Kazemi et al. [227] reported that, in an 8-week, randomised, double-blind trial, supplementation with *L. helveticus* R0052 and *B. longum* R0175 (1 × 10^10^ CFU/mL) substantially reduced BDI scores from 17.39 to 9.1 relative to the placebo. Zhang et al. [221] demonstrated that daily supplementation with *L. paracasei* Shirota (LcS) for 9 weeks significantly improved depressive symptoms in patients with depression. Another study by Moludi et al. [228] showed that an 8-week course of *L. rhamnosus* GG alleviated symptoms of chronic inflammation, depression, and anxiety in patients with coronary artery disease (CAD). Steenbergen et al. [229] conducted a study involving 40 healthy students who received a probiotic containing *B. bifidum* W23, *B. lactis* W52, *L. acidophilus* W37, *L. brevis* W63, *L. casei* W56, *L. salivarius* W24, *L. lactis* W19, and W58, or a placebo, for 28 days. The probiotic reduced negative cognitive responses to low mood, particularly rumination and aggressive thoughts. Another randomised, triple-blind, placebo-controlled trial with 71 participants used a similar probiotic formulation for 8 weeks [230]. The multi-strain probiotic, including *B. bifidum* W23, *B. lactis* W51 and W52, *L. casei* W56, *L. salivarius* W24, *L. lactis* W19 and W58*, L. brevis* W63, and W37, was evaluated for its effects on individuals with varying severity of depression. Results showed a significant reduction in cognitive reactivity and improvements in depressive symptoms, as measured by the BDI and Beck Anxiety Inventory (BAI). A 12-week, randomised controlled trial by Kim et al. [231] found that probiotic supplementation containing *B. bifidum* BGN4 and *B. longum* BORI notably decreased pro-inflammatory bacteria in the gut and increased serum BDNF levels in elderly participants. Moschonis et al. [232] reported that a multi-strain probiotic comprising *L. fermentum* LF16, *L. rhamnosus* LR06, *L. plantarum* LP01, and *B. longum* BL04, taken daily for 12 weeks, yielded beneficial effects in adults with subthreshold depression. Schaub et al. [233] examined the impact of a probiotic blend containing eight strains, including *S. thermophilus* NCIMB 30438, *B. brevis* NCIMB 30441, *B. longum* NCIMB 30436, *L. acidophilus* NCIMB 30442, *L. plantarum* NCIMB 30437, *L. paracasei* NCIMB 30439, and *L. delbrueckii* subsp. *bulgaricus* NCIMB 30440, on neuronal processes and microbiome alterations in 60 patients experiencing depressive episodes. Following treatment, those receiving probiotics exhibited a greater reduction in Hamilton Rating Scale for Depression (HAM-D) scores, accompanied by a concomitant increase in *Lactobacillus* abundance and alleviation of depressive symptoms. In clinical studies on academic stress, an 8-week course of L. casei Shirota (1 × 109 CFU/mL) demonstrated benefits, including reductions in cortisol and anxiety among students undergoing therapy [234]. Another study found that 8 weeks of daily *L. casei* Shirota supplementation led to notable reductions in anxiety, covering both cognitive and physical symptoms, as well as perceived stress among athletes [235]. Zhu et al. [236] also observed that a twice-daily intake of *L. plantarum* JYLP-326 for 3 weeks among university students significantly reduced anxiety, depression, and insomnia, and partially restored gut microbiota composition.

A summary of selected trials investigating the use of probiotic strains with psychobiotic potential is presented in Table 3.

**Table 3:** Selected clinical studies on the effects of psychobiotics on psychiatric symptoms and the central nervous system function.

Psychobiotic	Dose	Type of Study	Number of Participants	Duration	Evaluated Outcome	Reference
*Lactiplantibacillus plantarum* JYLP-326	1 g of lyophilized JYLP-326 powder at a fixed dosage of 1.5 × 10^10^ CFU		60 students with depression, anxiety and sleeping problems	3 weeks	Alleviated anxiety and depression	[236]
*Lactobacillus acidophilus* LA-5 *Lacticaseibacillus paracasei* L. CASEI-01	Cultured milk drinks containing 10^9^ CFU		110 participants with IBS and depression	12 weeks	Reduced depression (both groups); increased serotonin (probiotic only)	[237]
*Bifidobacterium animalis* subsp. *lactis* LMG P-21384 [BS01] *Bifidobacterium breve* DSM 16604 [BR03] *Bifidobacterium longum* DSM 16603 [BL04] *Lacticaseibacillus rhamnosus* ATCC 53103 [GG]	21,384–2.50 × 10^10^ CFU/dose, 16,604–1.00 × 10^10^ cfu/dose, 16,603–8.00 × 10^9^ cfu/dose, 53,103–4.50 × 10^10^ CFU/dose	Randomized, double- masked, and placebo-controlled trial	266 chemotherapy patients post-surgery for gastrointestinal cancer	4 weeks	Improvement in probiotic groups: depression 60.4%, anxiety by 57.0%, stress by 60.4%. Placebo group: deterioration in all parameters	[238]
*Lactiplantibacillus plantarum* Lp815	1 or 5 billion CFU/day		105 participants with mild or severe anxiety	6 weeks	Improvement in more than one anxiety category: 26% in placebo, to 37% with 1 billion CFU, to 68% with 5 billion CFU.	[239]
*Levilactobacillus brevis* P30021 *Lactiplantibacillus plantarum* P30025	>2 × 10^9^ CFU/day	Randomized, double-blind, placebo-controlled, crossover study	Probiotic group: 44 adults Placebo group: 43 adults	12 weeks	Reduced depressive symptoms and rumination; no effect on subjective stress; increased probiotic abundance in responders	[201]
*Streptococcus thermophilus* NCIMB 30438 *Bifidobacterium breve* NCIMB 30441 *Bifidobacterium longum* NCIMB 30435 *Bifidobacterium infantis* NCIMB 30436 *Lactobacillus acidophilus* NCIMB 30442 *Lactiplantibacillus plantarum* NCIMB 30437 *Lacticaseibacillus paracasei* NCIMB 30439 *Lactobacillus delbrueckii* subsp*. bulgaricus* NCIMB 30440	9 × 10^10^ colony-forming units (CFU)/g bifidobacteria, 8 × 10^10^ lactobacilli, and 20 × 10^10^ of *S. salivarius* subsp. *thermophilus,* resulting in a daily dose of 900 billion CFU/d		60 patients with major depressive disorder (MDD)	4 weeks	Improved immediate memory and hippocampal function; increased hippocampal activation during working memory tasks	[240]
*Bifidobacterium breve* CCFM1025 *Bifidobacterium longum* CCFM687 *Pediococcus acidilactici* CCFM6432	4 × 10^9^ CFU/g each strain	Two-arm parallel design, placebo-controlled, double-blinded, randomised controlled trial	28 MDD patients	4 weeks	Reduced depressive symptoms; improved gastrointestinal function; modulation of the serotonergic system	[217]
Sanprobi Barrier: *Bifidobacterium bifidum* W23 *Bifidobacterium lactis* W51 *Bifidobacterium lactis* W52 *Lactobacillus acidophilus* W37 *Levilactobacillus brevis* W63 *Lacticaseibacillus casei* W56 *Ligilactobacillus salivarius* W24 *Lactococcus lactis* W19 *Lactococcus lactis* W58	4 capsules (2 × 10^9^ CFU)/day	Randomized Double-Blind Placebo-Controlled Pilot Study	38 patients after bariatric surgery with depressive symptoms	5 weeks	No significant differences after probiotic therapy	[241]
*Bacillus subtilis* *Bifidobacterium bifidum* *Bifidobacterium breve* *Bifidobacterium infantis* *Bifidobacterium longum* *Lactobacillus acidophilus* *Lactobacillus delbrueckii* subsp*. bulgaricus* *Lacticaseibacillus casei* *Lactiplantibacillus plantarum* *Lacticaseibacillus rhamnosus* *Lactobacillus helveticus* *Ligilactobacillus salivarius* *Lactococcus lactis* *Streptococcus thermophilus*	4 capsules daily of probiotic (2 × 10^9^ colony-forming units per capsule)	Single-centre, double-blind, placebo-controlled pilot randomized clinical trial	49 patients with MDD showing incomplete response to antidepressant treatment	8 weeks	No significant changes after probiotics treatment	[242]
*Limosilactobacillus reuteri* *Lactobacillus acidophilus* *Limosilactobacillus fermentum* *Bifidobacterium bifidum*	Powdered probiotics in a sachet, taken orally (2 × 10^9^ CFU each)	Double-blinded, placebo-controlled, randomized trial	34 children aged 8–12 years old with a diagnosis of ADHD	8 weeks	Decreased ADHD-RS (β −3.31, *p* = 0.006) and HAM-A (β −1.91, *p* = 0.01) scores in the probiotic group, along with reduced serum high-sensitivity C-reactive protein (hs-CRP) and increased plasma total antioxidant capacity (TAC).	[243]
*Bifidobacterium bifidum* W23 *Bifidobacterium lactis* W51 *Lactobacillus acidophilus* W22 *Lacticaseibacillus casei* W56 *Lacticaseibacillus paracasei* W20 *Lactiplantibacillus plantarum* W62 *Ligilactobacillus salivarius* W24 *Lactococcus lactis* W19	Orally administered as a drink (7.5 × 10^9^ CFU in total)	Double-blinded placebo-controlled randomized trail	61 inpatients with major depressive disorder (MDD)	4 weeks	Decrease in IL-6 gene expression in the treated group	[244]
NVP-1704 probiotic: *Limosilactobacillus reuteri* NK33 *Bifidobacterium adolescentis* NK98	500 mg capsule contained 2.5 × 10^9^ colony-forming units of microorganisms (2.0 × 10^9^ CFU for *Lactobacillus reuteri* NK33), (0.5 × 10^9^ CFU for *Bifidobacterium adolescentis* NK98).	Double-blind randomized, placebo-controlled trial	177 healthy adults with subclinical symptoms of depression, anxiety, insomnia	8 weeks	Reduced depression (weeks 4 and 8) and anxiety (week 4), improved sleep quality, and decreased serum IL-6 levels in the treated group.	[245]

In summary, accumulating evidence from in vitro studies, animal models, and clinical research suggests that psychobiotics, particularly strains of *Lactobacillus* and *Bifidobacterium*, may be adjunctive strategies for managing mental health disorders [211,215,217,237,245]. In vitro studies have demonstrated that selected probiotic strains can produce neuroactive compounds, modulate inflammatory signalling, and influence neurotransmitter-related pathways involved in MGB axis communication. Animal studies further support these findings, showing that psychobiotic administration may affect stress responsiveness, GABAergic signalling, neuroinflammation, and behaviour associated with anxiety and depression-like phenotypes [209,210]. However, translating these promising preclinical findings into human clinical outcomes remains inconsistent.

Clinical studies have produced mixed results, and the field continues to face substantial methodological and translational challenges [246]. Considerable heterogeneity exists among studies with respect to probiotic strains, dosages, intervention duration, study populations, and assessed endpoints, limiting the comparability and generalizability of findings [145,247]. A meta-analysis conducted by Kadosh et al. [248] reported mixed effects of psychobiotic interventions on stress and anxiety in young people. Although some strains, such as *L. casei* Shirota and *B. bifidum*, were associated with reduced salivary cortisol levels and lower self-reported stress during examination periods, most studies did not demonstrate significant reductions in anxiety symptoms. Furthermore, some studies reported potential adverse effects, including increased anxiety scores [249] or elevated heart rate [250] following probiotic supplementation.

The translational gap between animal and human studies was highlighted by Kelly et al. [251], who demonstrated that *L. rhamnosus* (JB-1), despite producing anxiolytic effects in mice, failed to significantly improve stress-related or cognitive outcomes in healthy human volunteers. Nevertheless, broader evidence remains encouraging. A comprehensive umbrella review by Du et al. [252], including 47 meta-analyses across 12 categories of neuropsychiatric outcomes, concluded that probiotic supplementation may exert beneficial effects on depressive symptoms, stress-related parameters, and cognitive function. However, the authors also emphasized substantial heterogeneity between studies, limited mechanistic evidence, and the need for further well-designed preclinical and clinical investigations to establish causal relationships and clarify the underlying biological mechanisms.

## 7. Prospects and Conclusions

The growing body of evidence highlights psychobiotics as a promising approach for modulating the MGB axis and supporting mental health. In particular, microbial production of bioactive metabolites, such as GABA, is a key mechanism by which probiotic strains may influence neuroendocrine signalling, immune responses, and gut barrier function. Developing fermented foods enriched with these metabolites offers a practical and accessible way to translate these findings into dietary interventions. Optimising fermentation processes and selecting high-performing strains have already enabled the production of functional foods with higher GABA levels. Evidence from experimental and clinical studies suggests that regular intake of GABA-enriched products may support mood regulation and enhance psychological well-being [253,254]. These findings support the idea of fermented foods as effective carriers of psychobiotic strains with multifunctional health-promoting properties. Despite these advances, several challenges remain. Variability in individual responses, limited standardisation of experimental protocols, and a lack of large-scale clinical data hinder the integration of psychobiotics into routine clinical practice. Future research should focus on well-designed, long-term human studies that encompass diverse populations and more effectively control for dietary variables to better understand the efficacy and mechanisms of action of psychobiotics.

Advances in bioinformatics, multi-omics approaches, and artificial intelligence are expected to play a crucial role in the future development of psychobiotics. These tools will facilitate accurate characterisation of microbial strains, identification of functional metabolites, and the design of personalised microbiome-based interventions. At the same time, technological improvements are essential to enhance strain stability, viability, and targeted delivery within the GT. In this context, integrating psychobiotics into personalised nutrition strategies presents a promising pathway for early intervention and prevention of neuropsychiatric and neurodegenerative disorders. However, their successful implementation will require a multidisciplinary approach combining microbiology, nutrition, clinical science, and systems biology.

In conclusion, psychobiotics, particularly GABA-producing strains, hold significant potential as functional ingredients in future foods and therapeutic methods. Their future application will depend on rigorous scientific validation, technological progress, and the development of evidence-based, personalised strategies for microbiome modulation.

## Data Availability

No new data were created or analyzed in this study. Data sharing is not applicable.
